# Recurrence and Prognostic Value of Circulating Tumor Cells in Resectable Pancreatic Head Cancer: A Single Center Retrospective Study

**DOI:** 10.3389/fsurg.2022.832125

**Published:** 2022-04-06

**Authors:** Qiao Zhang, Feng Xia, Qiang Sun, Wenjing Cao, Ali Mo, Weiming He, Jiazhen Chen, Weiqiao Zhang, Weiqiang Chen

**Affiliations:** ^1^Guangdong Medical College, Zhanjiang, China; ^2^Department of Hepatic Surgery Center, Tongji Medical College, Tongji Hospital, Huazhong University of Science and Technology, Wuhan, China; ^3^Department of Hepatobiliary Surgery, Zhongshan Hospital Affiliated to Sun Yat-sen University, Zhongshan, China; ^4^Southern Medical University Graduate School, Guangzhou, China

**Keywords:** pancreatic cancer, pancreaticoduodenectomy, recurrence, prognosis, circulating tumor cells

## Abstract

**Background and Aim:**

To investigate the effect of preoperative circulation tumor cells (CTCs) on postoperative recurrence and overall survival prognosis of pancreatic head cancer after pancreaticoduodenectomy (PD).

**Methods:**

From March 2014 to January 2018, 73 patients with pancreatic head cancer underwent radical resection (R0) in Zhongshan People's Hospital. CTCs in peripheral blood of patients with pancreatic head cancer were detected by “Cyttel” method before PD. Seventy-three patients were divided into positive and negative groups according to the positive criteria. To explore the relationship between the clinical data of CTCs and disease-free survival (DFS) and overall survival (OS). Cox proportional hazards model was used to analyzing the risk factors affecting the postoperative recurrence and the survival prognosis of patients.

**Results:**

41 patients (56.2%) were in the CTC-positive group. Preoperative CTCs were correlated with tumor vascular invasion, CA199 level and postoperative liver metastasis (*P* < 0.05). Preoperative CTC-positive, lymph node metastasis, vascular invasion, and nerve invasion were independent risk factors for DFS (*P* < 0.05). Preoperative CTC-positive, tumor diameter > 2 cm and vascular invasion were independent risk factors for OS of patients (*P* < 0.05).

**Conclusion:**

The detection of CTCs before PD is an important factor affecting the DFS and OS of pancreatic head cancer, which is significant in guiding clinical work.

## Introduction

Pancreatic cancer is a common malignant tumor in the digestive system, which progresses rapidly because of its inconspicuous early clinical symptoms and high malignancy. Most patients are in the advanced stage when patients present with symptoms, with an inferior prognosis (1, 2). To date, pancreaticoduodenectomy (PD) is the mainstay of achieving long-term survival in patients with pancreatic head cancer (3–5). However, postoperative recurrence is a risk factor affecting the prognosis of patients, and the 5-year survival rate varies between 5 and 20% (2, 5, 6). Therefore, it is of great significance to predict the postoperative recurrence of pancreatic head cancer to improve the survival prognosis of patients. Past studies have shown that tumor size, lymph node metastasis, vascular invasion, nerve invasion, and the level of CA199 are independent risk factors for postoperative recurrence and survival prognosis of pancreatic head cancer (7–10). In addition, CTCs play an essential role in the progression of malignant tumors. Many literatures have shown that CTCs are associated with OS and DFS of many malignant tumors, especially breast cancer, colorectal cancer, and prostate cancer (11–14). Therefore, this study explore the correlation between CTCs in postoperative recurrence and pancreatic head cancer survival prognosis.

## Materials and Methods

### Patient Population

This study enrolled patients with pancreatic head cancer admitted to the Department of General Surgery I of Zhongshan People's Hospital from March 2014 to January 2018 and underwent PD treatment. Inclusion criteria (1) postoperative pathological diagnosis of pancreatic cancer; (2) detection of CTCs within 3 days before surgery; (3) no any neoadjuvant therapy; (4) radical resection (R0); (5) postoperative unified standard adjuvant chemotherapy; (6) with complete serological and imaging data; exclusion criteria: (1) patients younger than 18 years old; (2) the presence of adjacent organ invasion and distant metastasis; (3) patients died because of surgical complications during the perioperative period; (4) postoperative follow-up data were missing. This study was a retrospective clinical study reviewed by the Ethics Committee of Zhongshan People's Hospital and followed the Declaration of Helsinki.

### Data Collection

All patients underwent abdominal enhanced Computed tomography (CT) or Nuclear magnetic resonance imaging (MRI), chest CT or X-ray scan. Laboratory tests include blood routine, liver and kidney function, coagulation function, CA199, and other examinations. Patients basic data, such as gender, age, alanine aminotransferase (ALT), aspartate aminotransferase (AST), lactate dehydrogenase (LDH), creatinine (Cr), blood urea nitrogen (BUN), albumin (Alb), total bilirubin (TIBL), direct bilirubin (DIBL), international normalized ratio (INR), thrombin time (PT), maximum tumor diameter, and pathological grade, were collected.

### Surgical Resection

Seventy-three patients with pancreatic cancer underwent surgical treatment at our medical center, and an experienced surgical team did all processes. The surgical methods were based on preoperative imaging examination and intraoperative exploration, including open pancreaticoduodenectomy (OPD) in 51 patients (69.9%), laparoscopic pancreaticoduodenectomy (LPD) in 20 patients (27.4%), and open pylorus-preserving pancreaticoduodenectomy in 2 patients (2.7%). Among the 21 patients of vascular invasion, 17 patients underwent surgical resection and direct vascular anastomosis, and 4 patients underwent surgical resection and vascular reconstruction to ensure that the surgical margin was R0 resection. R0 resection was defined as the absence of residual tumor tissue of 1 mm within the resection margin of the surgical specimen macroscopically and microscopically.

### Analysis and Identification Methods of CTCs

Three days before surgery, we drew 5 ml of peripheral blood as a sample for inspection and strictly processed the sample according to the manufacturer's instructions. The “Cyttel” method (Jiangsu, China) identified the detection of CTCs, whose principles include negative immunomagnetic particle method and immunofluorescence *in situ* hybridization (im-FISH).

The former mainly uses immunomagnetic particles as the carrier, through the principle of antigen–antibody reaction, combined with centrifugation technology, to remove leukocytes from the blood *in vitro* to separate rare cells. Then, the samples were fixed on glass slides, dehydrated with ethanol, dried, and then hybridized with chromosome centromere probe No. 1 and chromosome centromere probe No. 8. Finally, 4-diamidine-2-phenylindole (DAPI) staining was added to seal the samples, and the CTCs were observed and counted under a fluorescence microscope (15, 16). It defined CTC count ≥1 as CTC-positive.

### Follow-Up

All patients were followed up throughout-patient service, telephone or WeChat. Follow up examination items included chest X-ray or chest CT scan, abdominal ultrasound, abdominal enhanced CT or MRI and PET-CT. They were followed up every 3 months for 2 years after surgery, from the day of surgery, and every 6 months after 2 years after surgery. Overall survival (OS) was defined as the time from surgery to patient death or last follow-up, and disease-free survival (DFS) was defined as the time from surgery to postoperative tumor recurrence or last follow-up. The cut-off date was July 1, 2021.

### Statistical Analysis

Continuous variables were expressed as mean ± standard deviation (SD), if they met normal distribution and had equal variance; the student's *t*-test was used to compare two groups. Continuous variables not meeting normal distribution and had equal variance were expressed as [median, interquartile range (IQR)], Kruskal-Wallis test was used for comparison between two groups; Categorical variables were reported as number (n) or percentages of patients (%). The χ2 test or Fisher's exact test compared categorical variables; Cox proportional hazards model was used for univariate and multivariate analysis; Kaplan-Meier method was used to measure DFS curve and OS curve. Log-rank test was used to compare DFS and OS between two groups; *P* < 0.05 was considered statistically significant. The above statistical analysis uses the R language (version 3.62). The main R package used is “tableone,” “survival” and “survminer” packages.

## Results

### Baseline Characteristics

This study collected 90 patients with pancreatic head cancer who underwent PD, and 73 patients (81.1%) underwent R0 resection. In the overall study population, 38 were male and 35 were female. The age range was 36–80 years, with a mean age of 62 years. The tumor diameter was between 1.2 and 5.0 cm, the mean tumor maximum diameter was 2.3 cm, 46 patients (63.0%) had tumors >2 cm in maximum diameter, and 38 patients (52.1%) had CA199 > 37 U/L. Lymph node metastasis was found in 38 patients (52.1%), vascular invasion in 21 patients (28.8%), and nerve invasion in 36 patients (49.3%). The clinicopathological data of the patients is shown in Supplementary Table 1.

### Relationship Between CTCs in Peripheral Blood and Clinical Data in Patients With Pancreatic Head Cancer

Peripheral blood CTCs were positive in 41 of 73 patients with pancreatic head cancer, ranging from 0 to 6 cells/3 mL, and preoperative CTCs positivity was significantly correlated with vascular invasion and preoperative CA199 (*P* < 0.05, Table 1). There was no statistical significance with clinical data such as gender, age, pathological grade, tumor size, lymph node metastasis, Cr, BUN, ALT, AST, Alb, TBIL, and DIBL (P > 0.05, Table 1). This suggests that preoperative CTCs are associated with tumor progression.

**Table 1:** Relationship between preoperative CTCs and basic clinicopathological characteristics of patients with pancreatic head cancer.

**Variable**	**CTC-negative (*n* = 32)**	**CTC-positive (*n* = 41)**	***P***
**Gender** (%)			0.308
Male	14.00 (43.75)	24.00 (58.54)	
Female	18.00 (56.25)	17.00 (41.46)	
Age (years mean[SD])	59.97 (9.12)	63.68 (7.91)	0.067
CA199 (U/L median [IQR])	19.45 [11.20, 44.70]	96.20 [8.40, 573.28]	<0.05
PT (s median [IQR])	11.45 [10.97, 11.90]	11.70 [11.30, 12.10]	0.247
INR (median [IQR])	1.00 [0.93, 1.05]	1.02 [0.97, 1.06]	0.245
FIB (g/L median [IQR])	3.25 [2.77, 4.38]	3.59 [3.10, 4.21]	0.685
ALT (U/L median [IQR])	44.00 [12.75, 109.28]	71.00 [20.00, 244.00]	0.061
AST (U/L median [IQR])	28.50 [17.50, 87.25]	65.00 [19.00, 143.00]	0.201
LDH (U/L median [IQR])	174.00 [144.00, 220.00]	185.00 [163.00, 232.00]	0.149
Alb (g/L median [IQR])	41.70 [37.00, 43.18]	41.30 [36.60, 43.70]	0.726
TIBL (umol/L median [IQR])	16.05 [10.10, 143.88]	91.30 [14.20, 199.80]	0.100
DIBL (umol/L median [IQR])	5.30 [3.80, 102.32]	44.60 [4.00, 145.20]	0.230
BUN (mmol/L median [IQR])	4.09 [3.39, 5.64]	4.20 [3.20, 5.40]	0.925
Cr (umol/L median [IQR])	61.50 [53.75, 80.25]	67.00 [55.00, 80.00]	0.697
Tumor diameter [cm mean (SD)]	2.18 (0.84)	2.47 (0.64)	0.096
**Pathological grade** (%)			0.203
Low	10.00 (31.25)	13.00 (31.71)	
Medium	7.00 (21.88)	16.00 (39.02)	
High	15.00 (46.88)	12.00 (29.27)	
**Vascular infiltration** (%)			<0.05
No	32.00 (100.00)	20.00 (48.78)	
Yes	0.00 (0.00)	21.00 (51.22)	
**Nerve invasion** (%)			0.122
No	20.00 (62.50)	17.00 (41.46)	
Yes	12.00 (37.50)	24.00 (58.54)	
**Metastases to lymph nodes** (%)			0.136
No	19.00 (59.38)	16.00 (39.02)	
Yes	13.00 (40.62)	25.00 (60.98)	

*PT, prothrombin time; INR, international normalized ratio; FIB, fibrinogen; ALT, alanine aminotransferase; AST, aspartate aminotransferase; LDH, lactate dehydrogenase; Alb, albumin; TIBL, total bilirubin; DIBL, direct bilirubin; BUN, urea nitrogen; Cr, creatinine; CTC, circulating tumor cell; SD, standard deviation; IQR, interquartile range*.

### Postoperative Recurrence of Pancreatic Head Cancer

All patients were followed up for an average of 14.8 months, ranging from 2 to 36 months. Fifty-nine patients had a recurrence, with a postoperative recurrence rate of 80.8% (59/73), most of which had recurrence at 1 year, with a recurrence rate of 65.8% (48/73) within 1 year. There were 17 patients of retroperitoneal recurrence alone and 40 patients of retroperitoneal recurrence with distant metastasis, including 24 patients of liver metastasis, 12 patients of peritoneal spread, 2 patients of pulmonary metastasis, 2 patients of spinal metastasis. In addition, 2 patients had liver metastases alone.

### Relationship Between CTCs and Postoperative Liver Metastasis

The mean CTCs was 2.7 in 26 patients with liver metastasis, 0.7 in the retroperitoneal metastasis group, and 1.0 in the retroperitoneal and peritoneal spread group after the operation.

**Figure 1:**
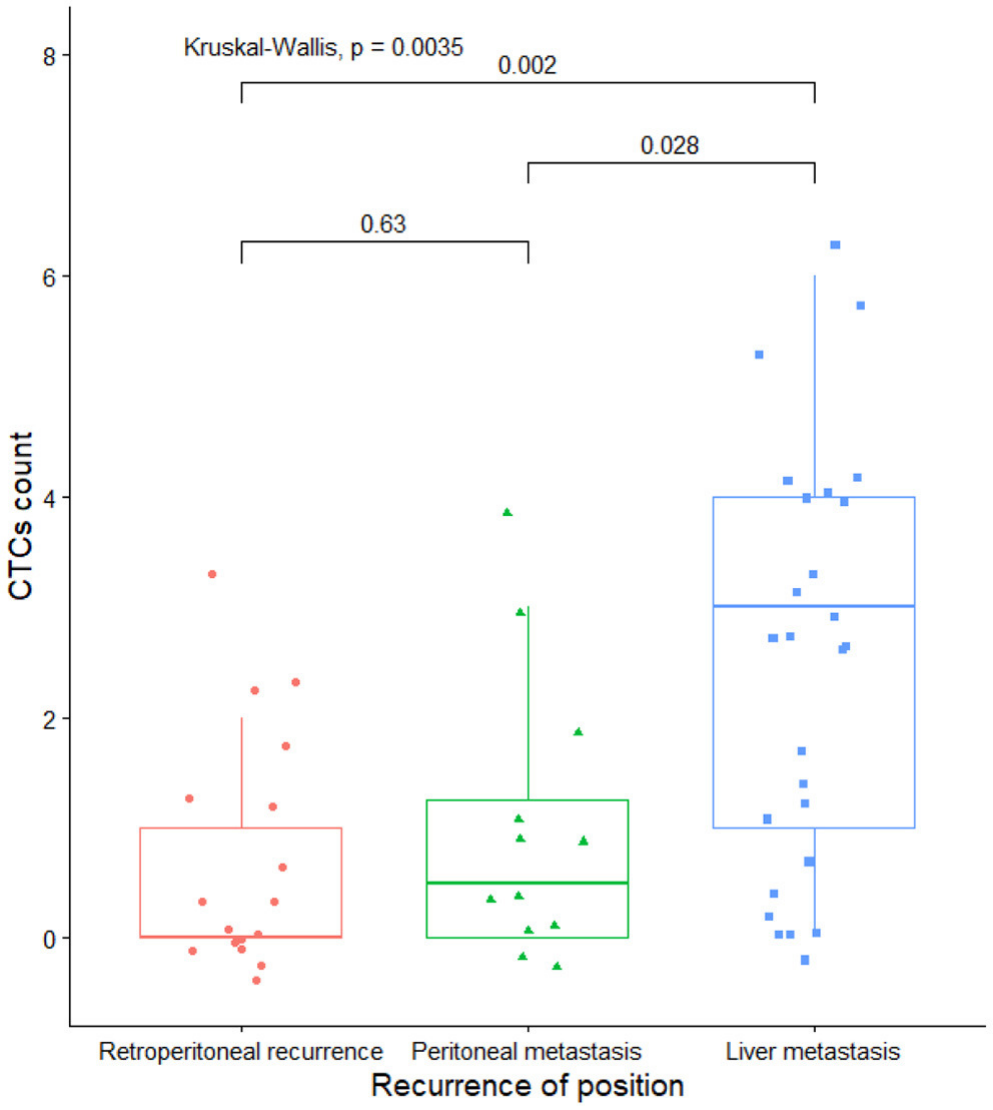
Relationship between CTCs content and the site of postoperative recurrence.

By Kruskal-Wallis test, the CTC in the group with liver metastasis was significantly higher than that in the retroperitoneal and peritoneal spread group (*P* < 0.05, Figure 1). There was no significant difference in the CTCs between the retroperitoneal and peritoneal spread groups (*P* > 0.05, Figure 1).

### Preoperative CTCs for Recurrence and Survival Prognosis of Patients With Pancreatic Head Cancer After Surgery

**Figure 2:**
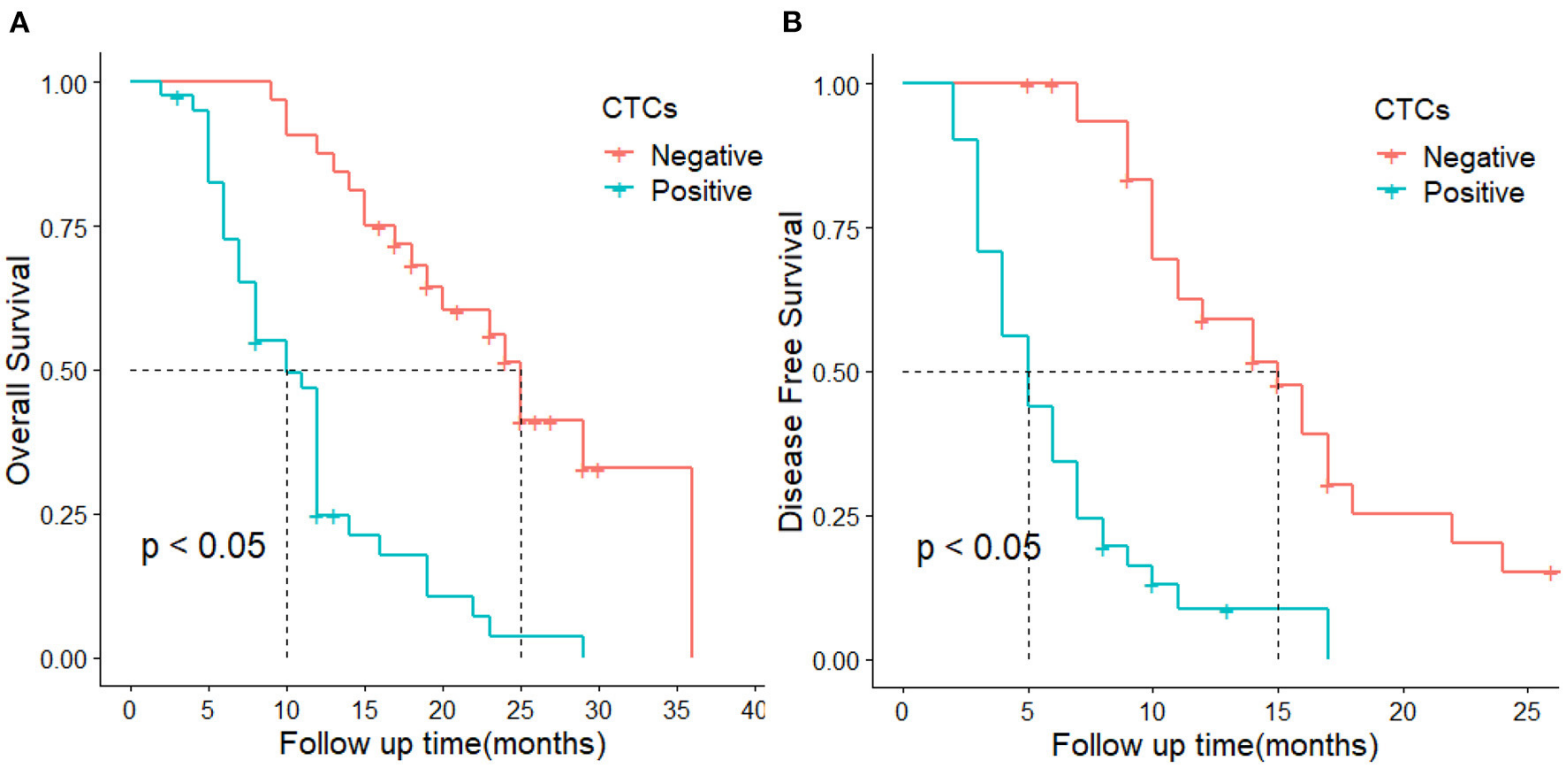
DFS and OS in CTC-positive and negative groups. **(A)** represents overall survival, **(B)** represent disease-free survival.

The median recurrence time was 5 months in patients with CTC-positive and 15 months in the CTC-negative group. The 1-year DFS rates were 59.2 and 7.8% in the CTC-negative and CTC-positive groups, respectively. The DFS of the CTC-positive group was significantly lower than that of the CTC-positive group. The difference was statistically significant (*P* < 0.05, Figure 2A). In terms of OS, the median survival time was 10 months and 25 months in the CTC-positive and CTC-negative group, respectively, and the 1-year survival rate was 87.5, 24.2% in the CTC-positive and CTC-negative group, respectively. The difference was statistically significant (*P* < 0.05, Figure 2B).

### Analysis of Independent Risk Factors of Postoperative Recurrence and Survival Prognosis

Univariate Cox analysis showed that CTC-positive, tumor size, lymph node metastasis, vascular invasion, nerve invasion, and preoperative CA199 > 37 U/L were prognosis factors for DFS (*P* < 0.05, Table 2), and multivariate Cox analysis suggested that CTC- positive, lymph node metastasis, vascular invasion, and nerve invasion were independent prognosis factors for DFS (*P* < 0.05, Table 2).

**Table 2:** Analysis of influencing factors of DFS of pancreatic head cancer.

	**Univariate analysis**		**Multivariate analysis**	
	**HR (95% CI)**	***P*-value**	**HR (95% CI)**	***P*-value**
Gender	0.676 (0.403–1.134)	>0.05		
Age	0.996 (0.967–1.026)	>0.05		
CA199 (>37 U/L)	1.890 (1.113–3.208)	<0.05	0.870 (0.466–1.626)	>0.05
PT	1.006 (0.924–1.095)	>0.05		
INR	1.092 (0.411–2.900)	>0.05		
FIB	1.098 (0.850–1.418)	>0.05		
ALT	1.000 (0.998–1.001)	>0.05		
AST	1.000 (0.998–1.002)	>0.05		
LDH	1.000 (0.996–1.005)	>0.05		
Alb	0.980 (0.924–1.038)	>0.05		
TIBL	1.001 (0.998–1.003)	>0.05		
DIBL	1.001 (0.998–1.004)	>0.05		
BUN	1.020 (0.992–1.049)	>0.05		
Cr	0.997 (0.983–1.010)	>0.05		
Tumor diameter >2 cm	2.668 (1.507–4.723)	<0.05	0.934 (0.446–1.955)	>0.05
CTC-positive	5.799 (3.158–10.649)	<0.05	4.172 (2.000–8.704)	<0.05
Low differentiation	1.123 (0.602–2.096)	>0.05		
Vascular invasion	6.931 (3.626–13.247)	<0.05	4.452 (1.934–10.244)	<0.05
Nerve invasion	2.212 (1.310–3.735)	<0.05	2.071 (1.073–3.996)	<0.05
Metastases to lymph nodes	1.951 (1.157–3.291)	<0.05	2.775 (1.563–4.928)	<0.05

*PT, prothrombin time; INR, international normalized ratio; FIB, fibrinogen; ALT, alanine aminotransferase; AST, aspartate aminotransferase; LDH, lactate dehydrogenase; Alb, albumin; TIBL, total bilirubin; DIBL, direct bilirubin; BUN, urea nitrogen; Cr, creatinine; CTC, circulating tumor cell; HR, hazard ratio*.

We explored which clinicopathological data affected the OS of patients. Univariate Cox analysis showed that CTC-positive, tumor size, vascular invasion, nerve invasion, and preoperative CA199 > 37U/L were risk factors for OS, and multivariate Cox analysis suggested that CTC-positive, tumor size and vascular invasion were independent risk factors for OS (*P* < 0.05, Table 3).

**Table 3:** Analysis of prognostic factors of postoperative survival of pancreatic head cancer.

	**Univariate analysis**		**Multivariate analysis**	
	**HR (95% CI)**	***P*-value**	**HR (95% CI)**	***P-*value**
Gender	0.826 (0.479–1.422)	>0.05		
Age	1.014 (0.981–1.047)	>0.05		
CA199 >37 U/L	2.568 (1.446–4.498)	<0.05	1.159 (0.588–2.283)	>0.05
PT	1.027 (0.948–1.113)	>0.05		
INR	1.376 (0.556–3.408)	>0.05		
FIB	1.159 (0.905–1.483)	>0.05		
ALT	1.001 (0.997–1.003)	>0.05		
AST	1.002 (1.000–1.004)	>0.05		
LDH	1.002 (0.997–1.007)	>0.05		
Alb	0.992 (0.935–1.052)	>0.05		
TIBL	1.002 (0.999–1.004)	>0.05		
DIBL	1.002 (0.999–1.005)	>0.05		
BUN	1.022 (0.996–1.049)	>0.05		
Cr	1.004 (0.991–1.018)	>0.05		
Tumor diameter >2cm	7.897 (3.737–16.691)	<0.05	4.077 (1.760–9.443)	<0.05
CTC-positive	5.290 (2.864–9.773)	<0.05	2.463 (1.180–5.139)	<0.05
Low differentiation	1.344 (0.691–2.611)	>0.05		
Vascular invasion	7.450 (3.984–13.930)	<0.05	2.421 (1.103–5.316)	<0.05
Nerve invasion	2.236 (1.289–3.880)	<0.05	1.478 (0.826–2.645)	>0.05
Metastases to lymph nodes	1.700 (0.970–2.981)	>0.05		

*PT, prothrombin time; INR, international normalized ratio; FIB, fibrinogen; ALT, alanine aminotransferase; AST, aspartate aminotransferase; LDH, lactate dehydrogenase; Alb, albumin; TIBL, total bilirubin; DIBL, direct bilirubin; BUN, urea nitrogen; Cr, creatinine; CTC, circulating tumor cell; HR, hazard ratio*.

## Discussion

Previous studies have shown that CTCs are tumor cells immersed in peripheral blood by malignant tumors as epithelial-mesenchymal transition (EMT). The immune system will recognize and remove most CTCs through cellular and humoral immunity, but a few CTCs can masquerade as normal cells to avoid immune surveillance and realize immune escape.

Tumor cells not monitored by the immune system play a vital role in the implantation, dissemination, and distant metastasis of malignant tumors by migration, adhesion, and other means, and are even closely related to the postoperative recurrence or even survival prognosis of patients (17, 18).

In recent years, some researches have gradually applied the detection of CTCs as a liquid biopsy technique to the study of postoperative recurrence and the survival prognosis of pancreatic cancer. Unfortunately, some studies have failed to achieve meaningful results, mainly due to: (1) Pancreatic cancer differs from other malignant tumors, with more interstitial components, relatively low tumor burden, and correspondingly fewer tumor cells flowing into the peripheral blood; (2) The venous return of the pancreas is not directly drained into the inferior vena cava to converge in the liver through the hepatic portal system; thus, this is also the reason the distant metastasis of pancreatic cancer is more likely to occur in the liver (19–23). Domestic and foreign studies have also shown that CTCs are related to pancreatic cancer invasion, and ultimately affect the postoperative recurrence and survival prognosis of pancreatic cancer (24, 25). Based on the debate, we used the “Cyttel” method to detect CTCs to explore their relationship with clinical features and the impact of postoperative recurrence and survival prognosis.

In the present study, the positive rate of preoperative CTCs in pancreatic head cancer was 56.1%, and the positive rate was roughly comparable with that reported in the past using nano microfluidic chip technology to detect CTCs in pancreatic cancer (24). But, it is lower than 64–73% of other gastrointestinal digestive malignancies (26). In order to solve the problem of the low detection rate of CTCs in pancreatic cancer caused by the return of pancreatic veins to the liver through the portal venous system, Wang et al. tried to directly extract portal vein blood to improve the detection rate of CTCs (27). Unfortunately, the detection rate of CTCs has not been effectively improved, and they believe that this is related to the lack of professional collection equipment and reagents for preserving samples, which also provides a lot of inspiration for our future research. In addition, Our research also found that the positive rate of peripheral blood CTCs detection in patients with postoperative liver metastasis was higher than that in patients with retroperitoneal local recurrence or peritoneal spread, and it was statistically significant. The results of this study have never been reported in past studies. Domestic scholar Liu's team carried out a relevant study on portal vein CTCs and liver metastasis of pancreatic cancer and found that it correlated portal vein CTCs with liver metastasis (28). Although the CTCs shed from the primary lesion pass through the filtering effect of the liver, a considerable number of CTCs can still reach the peripheral blood circulation. We can indirectly know the portal vein CTCs load by detecting peripheral blood CTCs, to predict the probability of postoperative liver metastasis better. The timely and effective removal or intervention of these so-called “metastases” of CTCs ultimately achieves the purpose of improving the postoperative survival of pancreatic cancer. The detection of CTCs from peripheral blood has great advantages over the detection of portal vein CTCs, which are manifested in: (1) the technique of obtaining CTCs from peripheral blood is easier to operate, the technical threshold is lower, and there is no need for the support of ultrasound, CT and other related equipment; (2) The operation of collecting CTCs through the portal vein is perilous. If there is a mistake in collecting portal blood, it may lead to the rupture of the portal vein and even endanger the patient's life. In summary, we believe CTC-positive associate with postoperative recurrence. The detection of CTCs in peripheral blood provides a brand-new indicator for clinical decision-making and has certain clinical value.

Firstly, considering that patients with CTC-positive are prone to recurrence after surgery, can we perform neo-adjuvant therapy in this part of patients to eliminate occult lesions in order to improve the DFS and OS (29). Secondly, the detection of CTCs in peripheral blood is helpful for the early detection of postoperative liver metastases. By strengthening postoperative monitoring of CTCs-positive patients, early detection of liver metastases and timely intervention of liver metastases (surgical resection or radiofrequency ablation) can be achieved, and to improve the long-term survival of patients (30).

Finally, our study also found that CTC-positive was correlated with vascular invasion, the concentration of high level of CA199, and not with clinicopathological variables such as age, tumor size, lymph node metastasis, nerve invasion, or pathological grade, which were the same as those reported in the past literature (24, 25, 31); As for the relationship with the preoperative CA199 level, a few scholars have reported (32). Of course, this needs to be confirmed by more studies in the future.

Our study also analyzed the clinicopathological variables associated with DFS and OS of patients using univariate and multivariate Cox proportional hazards models. CTC-positive, vascular invasion, nerve invasion, and lymph node metastasis are independent risk factors for postoperative recurrence, and the latter three variables have also been confirmed in past studies (33–38). CTC-positive, vascular invasion, and tumor size were independent risk factors affecting OS, which were also consistent with past reports (39, 40). The above results show that peripheral blood CTCs play a pivotal role in DFS and OS in patients with pancreatic cancer.

Of course, our study also has limitations: (1) The size of our study population is small, and we expect a larger population to verify our conclusions in the future; (2) Considering the high cost of CTCs detection, it cannot be used as a routine detection method, especially in economically backward areas. But we believe that with the improvement of detection methods, the cost of CTCs detection will be reduced. It will be more commonly used in clinical work.

## Conclusion

In conclusion, we believe the CTCs are related to the postoperative recurrence and survival prognosis of pancreatic head cancer, and can be used as an important indicator to evaluate the recurrence risk and clinical prognosis of pancreatic head cancer. We believe that the detection of CTCs will help to guide the clinical practice of pancreatic head cancer in the future.

## Data Availability Statement

The original contributions presented in the study are included in the article/Supplementary Material, further inquiries can be directed to the corresponding author.

## Author Contributions

QZ and FX wrote the manuscript. AM and QS provided the cases. AM, JC, and WZ provided the nuclear medical images and interpretation of the data. WCa provided data. WCh reviewed and edited the manuscript. All authors read and approved the manuscript.

## Conflict of Interest

The authors declare that the research was conducted in the absence of any commercial or financial relationships that could be construed as a potential conflict of interest.

## Publisher's Note

All claims expressed in this article are solely those of the authors and do not necessarily represent those of their affiliated organizations, or those of the publisher, the editors and the reviewers. Any product that may be evaluated in this article, or claim that may be made by its manufacturer, is not guaranteed or endorsed by the publisher.

## Abbreviations

PD: Pancreaticoduodenectomy
CT: Computed tomography
MRI: Nuclear magnetic resonance imaging
CTC: Circulating tumor cells
PT: Prothrombin time
INR: International normalized ratio
FIB: Fibrinogen
ALT: Alanine aminotransferase
AST: Aspartate aminotransferase
LDH: Lactate dehydrogenase
Alb: Albumin
TIBL: Total bilirubin
DIBL: Direct bilirubin
BUN: Urea nitrogen
Cr: Creatinine
HR: Hazard ratio
SD: Standard deviation
IQR: Interquartile range
OS: Overall survival
DFS: Disease-free survival
EMT: Epithelial-mesenchymal transition.
