# Supplementary material for: Recurrence and Prognostic Value of Circulating Tumor Cells in Resectable Pancreatic Head Cancer: A Single Center Retrospective Study
Source: Front Surg. 2022 Apr 6;9:832125. doi: 10.3389/fsurg.2022.832125 (PMC9019076; doi:10.3389/fsurg.2022.832125)
Supplement: Supplementary file 1 [file Table_1.DOCX]

Supplement Table 1. Baseline characteristics of the overall population

| **Variable** | Characteristics |
| --- | --- |
| **CTC**(median [IQR])） | 1.00 [0.00, 3.00] |
| **Age**( years mean[SD]） | 62.05 (8.60) |
| **CA199**(U/L median [IQR]) | 42.80 [9.69, 242.80] |
| **PT** (s median [IQR]) | 11.70 [11.20, 12.10] |
| **INR** (median [IQR]) | 1.02 [0.96, 1.06] |
| **FIB** (g/L median [IQR]) | 3.51 [3.00, 4.34] |
| **ALT** (U/L median [IQR]) | 54.00 [16.00, 160.00] |
| **AST** (U/L median [IQR]) | 43.00 [18.00, 124.00] |
| **LDH** (U/L median [IQR]) | 184.50 [154.00, 226.00] |
| **Alb** (g/L median [IQR]) | 41.70 [36.60, 43.60] |
| **TIBL** (umol/L median [IQR]) | 56.60 [10.80, 182.00] |
| **DIBL** (umol/L median [IQR]) | 21.20 [3.90, 141.10] |
| **BUN** (mmol/L median [IQR]) | 4.14 [3.30, 5.56] |
| **Cr** (umol/L median [IQR]) | 66.00 [54.00, 80.00] |
| **Tumor diameter** (cm mean (SD)) | 2,30 (1.26) |
| **Gender** |  |
| Male | 38 |
| Female | 35 |
| **Tumor diameter** (cm) |  |
| ≤2 | 27 |
| ＞2 | 46 |
| **Pathological grade** |  |
| Low | 23 |
| Medium | 23 |
| High | 27 |
| **Vascular Infiltration** |  |
| No | 52 |
| Yes | 21 |
| **Nerve invasion** |  |
| No | 37 |
| Yes | 36 |
| **Metastases to lymph nodes** |  |
| No | 35 |
| Yes | 38 |
| **CTC positive** |  |
| No | 32 |
| Yes | 41 |

***Abbreviation:*** *PT,* prothrombin time *INR* international normalized ratio*, FIB,* fibrinogen, *ALT* alanine aminotransferase, *AST* aspartate aminotransferase, *LDH,* lactate dehydrogenase, *Alb* albumin, *TIBL* total bilirubin, *DIBL* direct bilirubin, *BUN*, urea nitrogen, *Cr* creatinine. *CTC,* circulating tumor cell
